# The radiological outcomes of one-stage posterior-only hemivertebra resection and short segmental fusion for lumbosacral hemivertebra: a minimum of 5 years of follow-up

**DOI:** 10.1186/s13018-019-1482-5

**Published:** 2019-12-11

**Authors:** Yu Wang, Zhen Liu, Changzhi Du, Benlong Shi, Xu Sun, Bin Wang, Zezhang Zhu, Yong Qiu

**Affiliations:** 1 Department of Spine Surgery, Nanjing Drum Tower Hospital, Clinical College of Nanjing Medical University, Zhongshan Road 321, Nanjing, 210008 China; 2 Department of Spine Surgery, Nanjing Drum Tower Hospital, Medical School of Nanjing University, Zhongshan Road 321, Nanjing, 210008 China

**Keywords:** Congenital scoliosis, Lumbosacral, Hemivertebra resection, Posterior-only, Instrumentation, Fusion

## Abstract

**Background:**

Previous studies have reported favorable short-term outcomes after posterior-only hemivertebra resection and short fusion in patients with LSHV. However, there is a paucity of data evaluating the long-term outcomes following this procedure. The aim of the study is to evaluate the radiological outcomes following posterior-only hemivertebra resection and short fusion for the treatment of congenital scoliosis (CS) secondary to lumbosacral hemivertebra (LSHV) with a minimum of a 5-year follow-up.

**Methods:**

A total of 23 patients treated with one-stage posterior-only LSHV resection and short fusion with a minimum of a 5-year follow-up were reviewed. Radiographic parameters including the Cobb angles of the lumbosacral curve and compensatory curve, the upper instrumented vertebra (UIV) tilt, and trunk shift were measured. The complications were recorded accordingly.

**Results:**

The mean duration of follow-up was 88.6 ± 28.5 months, and the average age at surgery was 7.8 ± 3.5 years. Fusion levels averaged 3.0 ± 0.7 segments. The lumbosacral curve was corrected from 30.7 ± 10.4° to 6.7 ± 7.1° after surgery (*P* < 0.001), 7.3 ± 6.1° 2 years after surgery, and 8.1 ± 7.0° at the last follow-up. The compensatory curve was spontaneously corrected from 23.7 ± 9.4° before surgery to 8.3 ± 5.2° after surgery (*P* < 0.001). However, the angle slightly increased to 9.0 ± 4.8° 2 years after surgery and to 9.6 ± 6.4° at the last follow-up. Trunk shift was improved from 27.3 ± 8.6 mm before surgery to 11.7 ± 9.4 mm after surgery, and it decreased to 10.8 ± 8.2 mm 2 years after surgery and 10.4 ± 8.8 mm at the last follow-up. One patient experienced transient neurologic deficits after surgery. One patient was observed to have screw loosening at 1-year follow-up and received revision surgery.

**Conclusion:**

One-stage posterior-only hemivertebra resection with short fusion is an effective procedure for LSHV, and the correction can be well maintained during longitudinal follow-up. Great attention should be paid to the restoration of lumbosacral horizontalization.

## Introduction

Congenital scoliosis (CS) secondary to lumbosacral hemivertebra (LSHV) is a complicated spinal deformity in young children and adolescents [1–6]. From an anatomic and biomechanical view, the lumbosacral region serves as a transitional joint between the highly mobile lumbar spine and the immobile sacrum. Therefore, hemivertebra located in this region frequently results in significant coronal decompensation and a long compensatory curve above, which is reported to progress approximately 1° to 3° per year if not treated [1, 3, 4]. Because lumbosacral deformity does not respond effectively to bracing, early surgical management is frequently recommended for patients with demonstrated curve progression and coronal imbalance [3].

Previously, LSHV resection with instrumentation and fusion was performed via one-/two-stage combined anterior and posterior approaches [7–11]. However, the combined procedure is aggressive with a longer operating time and more blood loss [7, 12, 13]. With the substantial advances in posterior instrumentation, the posterior-only approach has gradually become preferred [12, 13]. However, there remains a debate regarding the selection of the most appropriate fusion level for this deformity, particularly in patients with immature skeletons [3, 4, 7]. Recently, short segmental fusion associated with posterior-only hemivertebra resection, due to its minimized side effects in terms of spine growth and mobility, has drawn the attention of spine surgeons [7, 12, 13].

Zhuang et al. [12] and Li et al. [13] demonstrated good correction of the lumbosacral curve and coronal trunk shift after posterior-only LSHV resection and short segmental fusion. However, both of the aforementioned studies were limited by a relatively short follow-up period; in particular, most of the enrolled patients were very young and far from skeletal maturity [12, 13]. How the compensatory curve and the coronal balance change during long-term follow-up remain unclear. Therefore, the current study was carried out to assess the radiographic outcomes with a minimum of 5 years of follow-up in LSHV patients who were treated with one-stage posterior-only hemivertebra resection and short segmental fusion and to evaluate the evolution of the compensatory curve and the coronal imbalance during long-term follow-up.

## Materials and methods

This retrospective study was approved by our hospital’s institutional review board. CS patients due to LSHV who were treated with hemivertebra excision between March 2003 and May 2013 were retrospectively reviewed. The inclusion criteria for this study were as follows: (1) having undergone one-stage posterior-only hemivertebra resection, (2) having had short segmental fusion (≤ 4 segments), and (3) having been followed up for at least 5 years. The exclusion criteria were those with multiple hemivertebrae, agenesis of the sacrum, history of spinal surgery, or unequal lengths of the lower extremities. Finally, 23 patients were recruited in our study.

### Surgical technique

After general anesthesia, the patient was placed in a prone position on a Jackson table, and a standard midline incision was performed. The posterior elements of the LSHV and the adjacent normal vertebrae that needed to be fused were carefully exposed. The pedicle screws were inserted into the adjacent normal vertebrae using the freehand technique. The posterior elements of the LSHV, including the facet joints, laminae, transverse processes, and posterior parts of the pedicle, were subsequently excised. After that, the lateral cortex of the LSHV was carefully exposed by blunt dissection. To stabilize the spine, a precontoured rod was provisionally screwed on the concave side and left unlocked. Then, the body of the hemivertebra was removed completely, followed by excision of the adjacent discs. Similarly, the contralateral facet and disc were also removed completely to obtain circumferential release. Cancellous bone from the hemivertebra was used for interbody fusion. Then, the convex side was gradually compressed to close the gap completely. In cases where a large gap was left due to the excision of a large hemivertebra, interbody fusion with a cage was used. During the whole procedure, the dural sac and the nerve roots were cautiously protected. The coronal balance and lumbosacral horizontality were checked under fluoroscopy before wound closure. All of the surgeries were performed under the neuromonitoring of sensory evoked potential (SEP) and motor evoked potential (MEP).

### Radiographic assessment

All radiographs were analyzed by two authors and the average values were calculated. Standing erect posteroanterior and lateral radiographs of the whole spine were evaluated before surgery, immediately after surgery, 2 years after surgery, and at the last follow-up. Three-dimensional computed tomography (CT) reconstruction was reviewed to identify the location and segmentation of the hemivertebrae preoperatively. Preoperative magnetic resonance imaging (MRI) of the whole spine was used to record the associated intraspinal malformations.

**Fig. 1:**
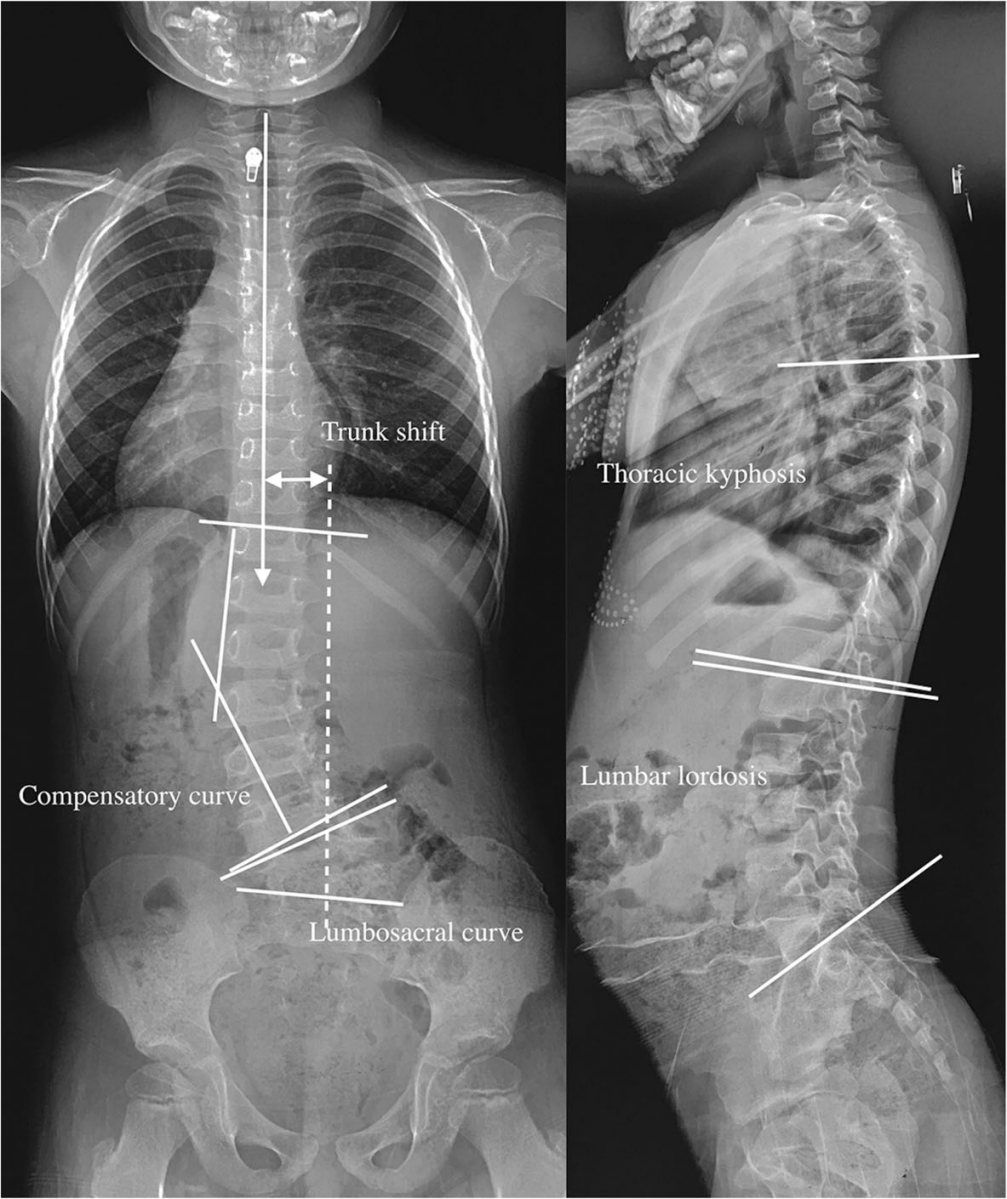
The coronal and sagittal parameters measured on standing whole spine X-rays

The parameters measured in the coronal plane included the Cobb angles of the lumbosacral and compensatory curves, the upper instrumented vertebra (UIV) tilt and trunk shift. Lumbosacral lordosis, lumbar lordosis, thoracic kyphosis, and the sagittal vertical axis (SVA) were measured in the sagittal plane. Lumbosacral scoliosis and lordosis were defined as the angle between the upper endplate of the vertebra above the hemivertebra and that of the sacrum [7]. The UIV tilt was measured as the angle between the superior endplate of the UIV and the horizontal line. Trunk shift was defined as the horizontal distance between the plumb line drawn from the middle of the C7 body and the central sacral vertical line (CSVL). Lumbar lordosis was measured as the angle between the superior endplate of L1 and the superior endplate of the sacrum. Thoracic kyphosis was defined as the angle between the superior endplate of T5 and the inferior endplate of T12 (Fig. 1).

### Statistical analysis

All parameters were analyzed with standardized statistical software (SPSS; version 22). Continuous values were described as the mean ± standard deviation. Parameters at different time periods were compared using the paired Student’s *t* test. Correlation analysis was used to identify the relationships between the decrement of UIV tilt and the improvement in trunk shift preoperatively to postoperatively immediately. A *P* value < 0.05 was regarded as statistically significant.

## Results

### Demographics

There were 14 boys and 9 girls with an average age of 7.8 ± 3.5 years (range, 2.5–13.0 years) at surgery. The hemivertebrae were located at L5 in 10 patients, L5–S1 in 11 patients, and L6–S1 in 2 patients. Among all of them, twenty-one (91.3%) patients were identified with a grade 0 Risser sign and the other 2 (8.7%) had grade 1 at presentation. The mean follow-up time was 88.6 ± 28.5 months (range, 60–156 months). At the last follow-up, 4 (17.4%) patients showed a grade 0 Risser, 2 (8.7%) were grade 1, 3 (13.0%) were grade 4, and 14 (60.9%) were grade 5.

Of the 23 patients, 11 patients were identified to have fully segmented hemivertebrae and 12 with semi-segmented hemivertebrae. The fusion span averaged 3.0 ± 0.7 levels (range, 2–4 levels), including 6 patients with 2 levels, 12 with 3 levels, and 5 with 4 levels. Interbody cage fusion was performed in 2 patients. The mean operation time was 196.7 ± 13.2 min (range, 175–230 min), and the average amount of blood loss was 271.7 ± 32.3 ml (range, 200–320 ml). One patient was identified with tethered cord and syringomyelia (Table 1).

**Table 1:** Demographic, anatomic and operative data of the 23 resected hemivertebrae

Patient no.	Sex	Age at surgery (years)	Location of hemivertebra	Fully/semi-segmented	United to	Associated intraspinal anomalies	Risser sign at surgery	Risser sign at last follow-up	Fusion segments	Operation time (min)	Blood loss (ml)	Cage	Follow-up (month)
1	M	11	L5–S1	Fully	–	–	0	5	3	210	290	–	156
2	F	4	L5–S1	Semi	L5	–	0	5	2	200	300	–	132
3	M	9	L5	Fully	–	–	0	5	4	230	320	–	72
4	M	10	L5–S1	Semi	L5	–	0	5	2	210	300	–	120
5	M	12	L5	Fully	–	–	0	5	3	195	280	–	96
6	F	8	L5	Fully	–	–	0	5	3	200	300	–	96
7	M	13	L5–S1	Semi	L4	–	1	5	3	210	300	–	96
8	M	9	L5–S1	Semi	L5	–	0	4	3	200	270	–	66
9	M	6	L5–S1	Fully	–	–	0	1	3	195	250	–	60
10	M	3	L5–S1	Semi	L5	–	0	5	3	180	280	–	132
11	F	11	L5	Semi	L4	–	0	5	4	200	320	–	60
12	M	11	L5–S1	Semi	L5	–	0	5	4	210	300	–	72
13	M	4	L5	Semi	L4	–	0	5	2	185	250	–	120
14	F	7	L5	Semi	L4	–	0	4	4	200	250	–	72
15	M	3	L5	Fully	–	–	0	0	4	200	300	–	96
16	F	3	L5	Fully	–	Tethered cord, syringomyelia	0	0	3	180	270	–	60
17	F	4	L6–S1	Semi	L6	–	0	1	3	185	290	–	96
18	M	12	L5–S1	Fully	–	–	0	5	3	180	250	–	92
19	F	5	L6–S1	Fully	–	–	0	4	2	200	230	–	104
20	F	6	L5–S1	Fully	–	–	0	0	2	180	220	–	60
21	M	13	L5	Semi	L4	–	1	5	3	210	250	Cage	60
22	F	10	L5–S1	Semi	L5	–	0	5	3	190	230	–	60
23	M	5	L5	Fully	–	–	0	0	2	175	200	Cage	60

### Correction results

**Fig. 2:**
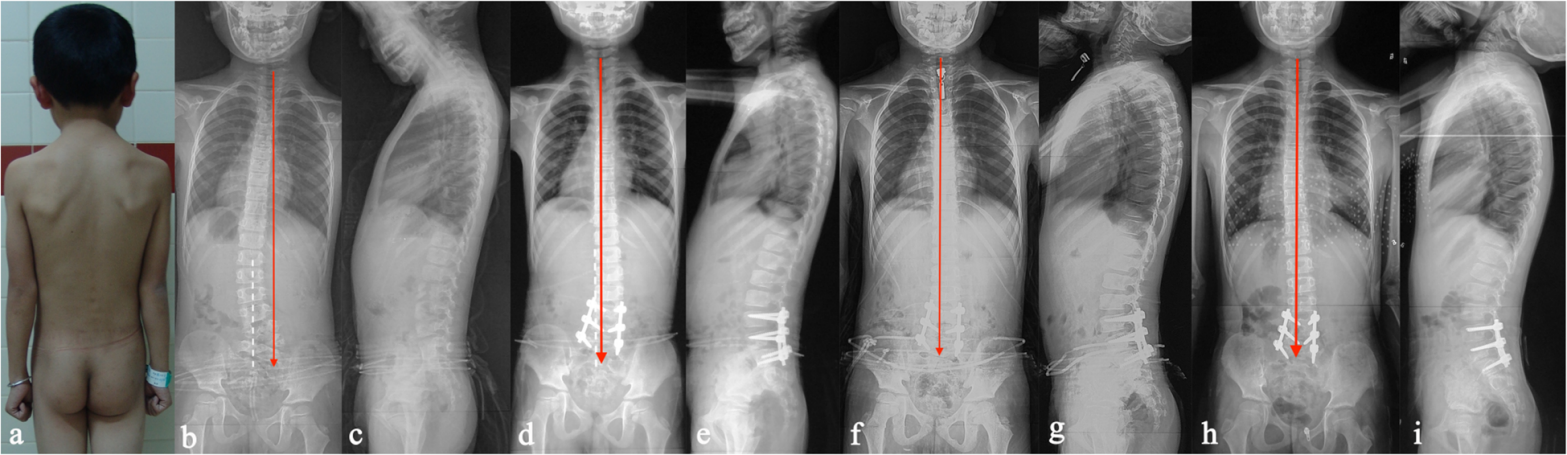
**a**–**c** A 6-year-old boy was identified with a lumbosacral hemivertebra located at L5–S1. The patient presented with a long proximal compensatory curve and significant coronal imbalance. **d**, **e** Postoperative radiographs showed excellent correction of both the local and compensatory curves and coronal imbalance. **f**, **g** The correction was well maintained at the 2-year follow-up. **h**, **i** Both the correction of scoliosis and trunk shift were well maintained at the 5-year follow-up

The lumbosacral curve averaged 30.7 ± 10.4° before surgery, 6.7 ± 7.1° (79.1% correction, *P* < 0.001) immediately after surgery, 7.3 ± 6.1° (76.9% correction) 2 years after surgery, and 8.1 ± 7.0° (73.3% correction) at the last follow-up. The UIV tilt significantly improved from 15.3 ± 6.4° before surgery to 3.8 ± 4.3° (*P* < 0.001) immediately after surgery, 4.0 ± 5.5° 2 years after surgery, and 4.6 ± 6.7° at the last follow-up. Accordingly, trunk shift was significantly improved from 27.3 ± 8.6 mm preoperatively to 11.7 ± 9.4 mm immediately postoperatively, 10.8 ± 8.2 mm 2 years postoperation, and 10.4 ± 8.8 mm at the last follow-up (Table 2, Fig. 2). Interestingly, the decrease in trunk shift was significantly correlated with the change in the UIV tilt (*r* = 0.615, *P* = 0.002).

**Table 2:** Comparisons of the coronal and sagittal parameters between pre-operation and postoperation

	Pre-op	Post-op	Correction rate (%)	*P* value (pre-op vs.post-op)	2 years post-op	Correction rate (%)	*P* value (post-op vs. 2 years post-op)	Last follow-up	Correction rate (%)	*P* value (post-op vs. last follow-up)
Coronal plane										
Lumbosacral curve (°)	30.7 ± 10.4	6.7 ± 7.1	79.1 ± 19.4	< 0.001	7.3 ± 6.1	76.9 ± 17.2	0.185	8.1 ± 7.0	73.3 ± 23.0	0.119
Compensatory curve (°)	23.7 ± 9.4	8.3 ± 5.2	64.2 ± 21.3	< 0.001	9.0 ± 4.8	59.7 ± 23.2	0.439	9.6 ± 6.4	60.1 ± 24.4	0.228
UIV tilt (°)	15.3 ± 6.4	3.8 ± 4.3	73.1 ± 28.1	< 0.001	4.0 ± 5.5	73.7 ± 30.6	0.583	4.6 ± 6.7	70.3 ± 35.0	0.145
Trunk shift (mm)	27.3 ± 8.6	11.7 ± 9.4	54.4 ± 35.8	< 0.001	10.8 ± 8.2	57.9 ± 31.6	0.219	10.4 ± 8.8	60.1 ± 31.4	0.360
Sagittal plane										
Lumbosacral lordosis (°)	16.4 ± 10.1	15.2 ± 7.0	–	0.475	16.2 ± 7.0	–	0.152	15.9 ± 7.3	–	0.484
Lumbar lordosis (°)	36.7 ± 13.4	35.9 ± 8.6	–	0.726	36.9 ± 6.5	–	0.464	38.8 ± 10.5	–	0.185
Thoracic kyphosis (°)	17.7 ± 10.3	17.6 ± 6.8	–	0.984	18.1 ± 4.8	–	0.622	16.7 ± 6.0	–	0.567
SVA (mm)	− 5.5 ± 15.0	− 9.2 ± 26.1	–	0.526	− 5.8 ± 20.7	–	0.225	− 8.5 ± 22.5	–	0.855

*UIV* upper instrumented vertebra, *SVA* sagittal vertical axis

The Cobb angle of the proximal compensatory curve was spontaneously corrected from 23.7 ± 9.4° before surgery to 8.3 ± 5.2° immediately after surgery, with an average correction rate of 64.2 ± 21.3% (*P* < 0.001). However, the angle slightly increased to 9.0 ± 4.8° 2 years after surgery and to 9.6 ± 6.4° at the last follow-up, although the difference was not significant. Progression (more than 5°) of the compensatory curve was observed in three patients. Brace treatment was prescribed, and no additional surgery was required due to compensatory curve progression at the last follow-up.

### Complications

Transient neurologic complications were observed in one patient. After 3 months of conservative treatment, this patient recovered completely. No infection, instrumentation breakage, or pseudoarthrosis was observed during the long-term follow-up. Another patient encountered S1 screw loosening on the right side at the 1-year follow-up and eventually received revision surgery.

## Discussion

The natural history of CS has been well documented in previous studies [1, 2]. The severity of deformity greatly depends on the type and location of the hemivertebra, and curve progression is usually unavoidable for a fully segmented or semi-segmented hemivertebra [1, 2]. Because of the lack of motile and compensatory capacity below the sacrum, hemivertebrae located in the lumbosacral region might frequently lead to significant coronal trunk shift and a long proximal compensatory curve at an early age [1–4]. In addition, conservative treatment, including bracing and traction, has been shown to be ineffective [3, 4]. Therefore, early surgical intervention should be recommended for patients with rapid curve progression and significant trunk imbalance [1–7, 12, 13].

Recently, one-stage posterior-only hemivertebra resection with short segmental instrumentation has become a popular treatment for young patients with LSHV due to the intrinsic benefit of preserving spinal growth and mobile segments [12, 13]. Zhuang et al. [12] reported that the lumbosacral curve had an 83% correction immediately after surgery and an 87% correction at the last follow-up. In another study by Li et al. [13], the correction rate was 65.5% immediately after surgery and 55.2% at the last follow-up. Consistent with the aforementioned studies [7, 12, 13], our results showed that the primary lumbosacral curve had a 79.1% correction immediately after surgery and a 76.9% correction at the 2-year follow-up. Additionally, the long-term outcomes demonstrated that posterior-only hemivertebra resection with short segmental fusion is an effective surgical procedure for young patients with LSHV.

Previous studies have confirmed that young age is a potential risk factor for curve progression [1, 2]. For skeletally immature patients receiving LSHV resection and short fusion, the evolution of the unfused proximal compensatory curve and coronal imbalance are the two major concerns. However, there has been a paucity of studies focusing on the evolution of the compensatory curve during long-term follow-up periods. Our results showed a 64.2% spontaneous correction of the proximal compensatory curve and a 54.4% correction of trunk shift immediately after surgery. In addition, with a minimum of 5 years of follow-up, 73.9% of patients reached or approached skeletal maturity (Risser grade 4 or 5). At the last follow-up, the compensatory curve displayed a 60.1% correction, and trunk shift displayed a 60.1% improvement, suggesting the long-term efficacy of the surgical technique.

The relationship between postoperative UIV tilt and surgical outcomes has been explored in scoliosis patients receiving correction surgery [14, 15]. Liu et al. [14] reported that UIV tilt was correlated with postoperative coronal imbalance in adolescent idiopathic scoliosis patients with Lenke 5C type curves. In adult degenerative scoliosis patients, Bao et al. [15] also found that patients with unsatisfactory postoperative lumbosacral horizontalization were at high risk of coronal imbalance. Similarly, in CS patients with LSHV, our study also showed that the improvement in trunk shift was significantly correlated with the correction of the UIV tilt, emphasizing the importance of a horizontal lumbosacral foundation for the restoration of coronal balance.

Failure to horizontalize UIV may be attributed to incomplete removal of the hemivertebrae or insufficient resection of the contralateral facet joint or disc. Incomplete removal of hemivertebrae may hamper convex compression. Moreover, the growth of the residual part of the hemivertebra may cause the lumbar “take-off” phenomenon and subsequent coronal imbalance, as demonstrated by Nakamura et al. [10]. In addition, the concave facet joint and disc should also be resected sufficiently to provide a circumferential release for the restoration of lumbosacral horizontalization. In patients with a large cavity after excision of a large hemivertebra, it may be difficult to completely achieve “bone to bone” closing via a simple compression maneuver. Therefore, a cage filled with a cancellous autograft may be used when necessary.

The neurologic complications of hemivertebra resection via the posterior approach have been well documented in previous studies [7, 13, 16, 17]. Bollini et al. [7] reported a tibialis motor deficit with incomplete recovery in a patient undergoing LSHV resection. Li et al. [13] noted that three patients encountered transient neurological complications after posterior-only LSHV resection. In our study, one patient with a semi-segmented hemivertebra located at L5–S1 was noted with transient right foot weakness after surgery, possibly due to the stretching of the nerve root. The patient recovered completely 3 months later. Hence, despite the high demand for this technique, LSHV resection via the posterior-only approach seems to be a safe procedure.

Implant-related failure is another major complication for young patients. Lyu et al. [18] reported two patients with implant failure in 17 CS patients with lumbosacral fixation. In the study by Ruf et al. [19], implant failure occurred in 7.3% (3/41) of patients. In our study, one patient was demonstrated to have screw loosening at the 1-year follow-up. Most patients with implant-related failures require revision surgery. The causes for this complication are manifold and include young age, limited implant, and short fusion [20]. Therefore, for young patients who undergo LSHV resection and short segmental fusion, immobilization via a brace for at least 3 months is indispensable.

To the best of our knowledge, this is the first study presenting the evolution of scoliosis and coronal imbalance with a long-term follow-up. Another highlight is that the homogeneous nature of this cohort, which excluded patients with multiple hemivertebrae, made our results more convincing. Despite the relatively small sample size, however, this study included the largest cohort to date. Furthermore, considering the harmfulness of excessive radiation exposure, a postoperative CT scan was not routinely performed in our study. Inevitably, another intrinsic weakness of this study is its retrospective nature. Additionally, the clinical outcomes were not assessed due to the relatively young age of the included cohort.

## Conclusions

For patients with CS due to LSHV, one-stage posterior-only hemivertebra resection and short segmental fusion can provide excellent scoliosis correction and trunk shift improvement. In addition, the restoration of lumbosacral horizontalization should be given great attention during surgery.

## Data Availability

Please contact author for data requests.

## Abbreviations

CS: Congenital scoliosis
CSVL: Central sacral vertical line
CT: Computed tomography
LSHV: Lumbosacral hemivertebra
MEP: Motor evoked potential
MRI: Magnetic resonance imaging
SEP: Sensory evoked potential
SVA: Sagittal vertical axis
UIV: Upper instrumented vertebra
